# Artificial Intelligence in Asthma and COPD: Current Status and Future Potential

**DOI:** 10.3390/jcm15062445

**Published:** 2026-03-23

**Authors:** Federica Marrelli, Chiara Lupia, Saverio Nucera, Daniela Pastore, Paolo Zaffino, Carolina Muscoli, Girolamo Pelaia, Corrado Pelaia

**Affiliations:** 1 Department of Health Sciences, “Magna Graecia” University—Catanzaro, 88100 Catanzaro, Italy; federicamarrelli1@gmail.com (F.M.); c.lupia@unicz.it (C.L.); saverio.nucera@unicz.it (S.N.); danielapastore11@gmail.com (D.P.); muscoli@unicz.it (C.M.); pelaia@unicz.it (G.P.); 2 Department of Experimental and Clinical Medicine, “Magna Graecia” University—Catanzaro, 88100 Catanzaro, Italy; p.zaffino@unicz.it; 3 Department of Medical and Surgical Sciences, “Magna Graecia” University—Catanzaro, Viale Europa—Località Germaneto, 88100 Catanzaro, Italy

**Keywords:** asthma, COPD, machine learning, deep learning, artificial intelligence

## Abstract

Interest in artificial intelligence (AI) is rapidly growing. In healthcare, especially through machine learning and deep learning, AI is emerging as a promising tool to support the diagnosis, management, and prevention of lung diseases and to advance personalized care, although it requires large, well-structured datasets. Clinicians must learn how to integrate AI into routine practice for conditions such as asthma and chronic obstructive pulmonary disease (COPD), while ensuring patient safety and building trust in these tools. Chronic respiratory diseases are major global causes of morbidity and mortality and place a substantial burden on healthcare systems; among them, asthma and COPD are chronic disorders characterized by airway obstruction and inflammation. This review highlights the rapid advancement of AI, and it aims to explore the literature’s evidence of its applicability in controlling chronic respiratory disorders, particularly in asthma and COPD. We conducted a narrative literature review by searching ScienceDirect, PubMed, and Google Scholar for English-language studies on artificial intelligence applications in asthma and COPD and by screening the references of relevant articles. The reviewed literature suggests that AI-based approaches are being applied across the asthma–COPD spectrum to support diagnosis and phenotyping, improve risk stratification and prediction of clinically relevant outcomes, and enable more continuous monitoring using heterogeneous data sources (e.g., clinical records, imaging, and digital health data). AI-based tools are poised to support clinicians in asthma and COPD across diagnosis, phenotyping, and monitoring; however, their safe implementation in routine care will require robust validation, transparency, and governance to ensure reliability and patient safety.

## 1. Introduction

Artificial intelligence (AI) has gained widespread clinical utilization and opens new opportunities for prevention, early diagnosis, and prompt and appropriate therapeutic intervention. Research in chronic respiratory diseases (CRDs) is gradually adopting AI techniques, following the overall trend of implementation in healthcare-related studies. The term AI refers to the simulation of human intelligence by computer systems, or the use of a computer to mimic intelligent behavior with minimal human interaction; it encompasses functions such as reasoning, learning, language processing, and the display of knowledge or information. When combined with vast volumes of well-characterized data, AI can provide models that are expected to enhance clinical practice and lead to improved care delivery, particularly in the management of chronic diseases [1]. Over the last decade, the application of AI has garnered substantial interest in the field of lung diseases [2]. In the domain of CRD care, there is a growing necessity for early identification, accurate staging, and effective management strategies. AI can improve the efficiency and accessibility of high-quality healthcare. Consequently, AI has attracted considerable interest, especially for early detection of asthma, monitoring COPD progression in at-risk groups, and enhancing diagnoses [3]. AI and machine learning (ML) models are heavily reliant on accessible data, and the healthcare sector generates vast amounts of data that must be mined for underlying insights. Large and complex datasets containing multiple sources of data can be examined appropriately using modern AI/ML approaches [1]. AI may be able to reduce the global burden of asthma and COPD, as well as the morbidity and mortality rates associated with them.

## 2. Data Source and Study Selection

In this narrative review, we performed a structured literature search to identify studies investigating the application of artificial intelligence (AI) to the diagnosis, classification/phenotyping, monitoring, prognostication, and management of asthma and chronic obstructive pulmonary disease (COPD). We searched PubMed, ScienceDirect, and Google Scholar from database inception to 20 December 2025 using combinations of keywords related to the following: (i) respiratory diseases (“asthma”, “COPD”, “chronic respiratory disease”, “lung disease”); (ii) AI methods (“artificial intelligence”, “machine learning”, “deep learning”, “neural network”, “natural language processing”, “NLP”); and (iii) clinical tasks (“diagnosis”, “management”, “treatment”, “prognosis”, “monitor”, “prediction”, “exacerbation”, “stratification”, “phenotype”). Boolean operators and alternative spellings were used to broaden retrieval where appropriate. The search retrieved n = 142 records (plus n = 10 from reference screening); after de-duplication (n = 29), n = 123 titles/abstracts were screened and n = 35 full texts were assessed, yielding n = 27 included studies.

We included peer-reviewed articles that reported the development, validation, or clinical evaluation of AI/ML/DL/NLP approaches applied to asthma and/or COPD using clinical data (e.g., electronic health records), pulmonary function tests, imaging (e.g., CT/HRCT), wearable/sensor data, or non-invasive biomarkers. We excluded studies not focused on asthma/COPD (or not clearly related to chronic respiratory diseases), studies without a clear AI component, purely technical papers without clinical data or outcomes, animal-only studies, conference abstracts without full-text availability, and editorials/commentaries. When multiple papers reported overlapping cohorts or models, the most complete and up-to-date report was prioritized.

Titles and abstracts were screened for relevance, followed by full-text assessment of potentially eligible articles. Reference lists of included papers and relevant reviews were also screened to identify additional eligible studies. For each included study, we extracted key methodological and clinical features (study design and setting, population, sample size, data modality, AI technique, target outcome, validation strategy, including internal vs. external validation when available, main performance metrics, and reported limitations). Findings were synthesized qualitatively and organized by application domain to highlight current evidence, methodological gaps, and future directions.

Table 1 summarizes the key characteristics of the representative included studies.

## 3. Artificial Intelligence Techniques in Lung Disease Management

In this review, we use “AI” as an umbrella term that includes (i) machine learning (ML), which learns predictive relationships from structured data (e.g., logistic regression, random forest, gradient boosting) and is commonly applied to EHR-based risk prediction such as exacerbation forecasting in asthma; (ii) deep learning (DL), a subset of ML that uses multi-layer neural networks and excels in high-dimensional inputs such as CT imaging (e.g., CNN- or transformer-based models for emphysema quantification and COPD severity assessment); (iii) natural language processing (NLP), which extracts structured variables from free-text clinical notes (e.g., automated chart review for asthma prediction indices); and (iv) large language models (LLMs), a newer class of NLP models that can generate or summarize text and may support tasks such as clinical documentation assistance or structured extraction from unstructured records, although clinical deployment requires careful validation, governance, and bias monitoring.

**Figure 1:**
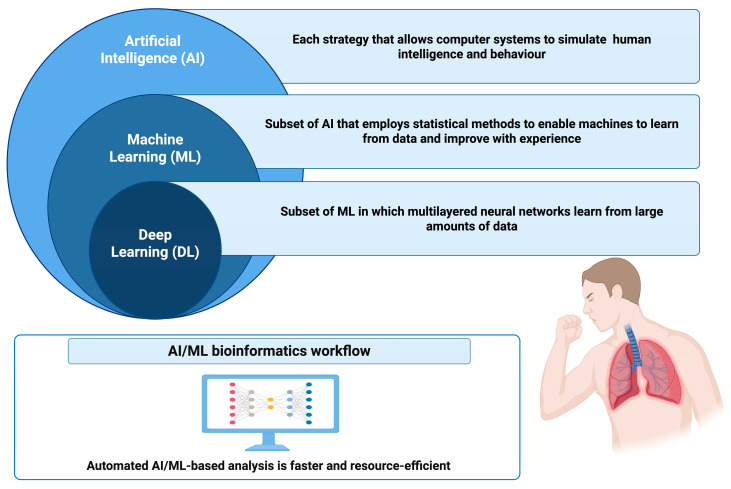
Relationship between Artificial Intelligence (AI), Machine Learning (ML), and Deep Learning (DL), as well as their role in data-driven clinical and bioinformatics workflows for faster and more efficient automated analyses. Created in BioRender. Marrelli, F. (2026) https://BioRender.com/ya9xdr9.

AI techniques that simulate human-like intelligence, including DL, ML and NLP, demonstrate their effectiveness in enhancing diagnostic accuracy and prognostic capabilities across lung diseases [2]. Figure 1 illustrates the relationship between AI, ML and DL.

ML and DL, sub-domains of AI, provide different features and applications in medical imaging. ML models generate predictions using algorithms that learn from data and often use structured datasets from medical imaging to discover trends and support diagnosis. ML includes supervised learning, in which models learn from labelled data to assist in tasks such as disease subtype classification and treatment response prediction; unsupervised learning, where patterns are identified without labelled data, which helps segment similar regions in CT images [12,13,14]. DL, an advanced subset of ML, employs multilayered neural networks. DL can learn from vast amounts of data, identifying intricate patterns and subtle changes that conventional image processing methods or expert humans could overlook [14,15]. Convolutional neural network (CNN) designs have significantly advanced DL and are utilized in various computer vision applications, including image classification, object detection, and semantic segmentation. The CNN identifies characteristics that vary from fundamental edges to intricate patterns [15,16]. Every design has unique characteristics and trade-offs regarding accuracy, computational complexity, and memory demands.

Understanding the design principles and performance attributes of these architectures is crucial for selecting the most suitable model for a specific task. Creating new CNN architectures is essential for choosing suitable models for specific tasks and for designing innovative CNN models tailored to domain-specific requirements, such as medical image analysis for characterizing and identifying COPD using CT scans [4,15,17,18,19]. Factors facilitating the rise of DL in imaging include massive, labelled datasets, advanced network architectures, and increasing computing capabilities. Both ML and DL offer advantages in medical imaging; however, the choice between the two depends on the specific requirements of the work. AI provides notable advancements in the field of radiology. Its integration not only improves diagnostic precision but also indicates a more patient-centered methodology, whereby personalized therapies and heightened safety become the norm. Generating high-quality images from lower-dose scans using AI techniques achieves a balance between patient safety and image clarity [20]. Moreover, predictive modelling is enabled by AI’s capacity to merge imaging data with electronic clinical information. This facilitates individualized patient management by providing insights into potential disease trajectories, therapeutic responses, or even complications [12]. As the volume of imaging performed in medicine continues to increase rapidly, AI will play an increasingly crucial role in image interpretation.

Accordingly, we organize the evidence below by clinical task (differential diagnosis, phenotyping, outcome prediction, imaging-based characterization, and remote monitoring), specifying the AI method and the data modality used in each study.

## 4. Asthma and COPD

### 4.1. Disease Burden and Artificial Intelligence Applications

Asthma and COPD are chronic respiratory disorders characterized by airflow limitation and inflammation and represent a major global burden. Asthma is a heterogeneous condition with variable respiratory symptoms and expiratory airflow obstruction that may resolve spontaneously or with treatment [21]. COPD is characterized by persistent respiratory symptoms (e.g., dyspnea, cough, sputum) and progressive airflow limitation related to airway and/or alveolar abnormalities. Globally, asthma and COPD account for a substantial share of chronic respiratory disease morbidity and mortality, as also highlighted by Global Burden of Disease estimates [22,23,24,25].

Asthma and COPD are recognized as heterogeneous conditions with distinct prognoses and treatment options, and making a clear distinction between them can be challenging in some patients due to overlapping clinical traits [21,24]. Pulmonary function tests remain central to diagnosis and severity assessment, requiring interpretation of ventilatory patterns and grading of airflow limitation according to international guidance [21,24]. In recent years, CT has become increasingly available in research and clinical practice, improving characterization of structural abnormalities, phenotypes, severity, and outcomes in asthma and COPD [21,24].

Because routine clinical data and spirometry are widely available, AI/ML models can support discrimination between asthma and COPD even with limited clinical variables [19]. For example, a decision support system using clinical factors (e.g., age, sex, sputum production, chest pain, smoking history) combined with spirometry has been proposed to detect and differentiate asthma and COPD [5]. These approaches are clinically relevant given that early stages may be managed in outpatient settings, whereas advanced disease and clinical worsening often require inpatient care [26,27]. Optimizing outpatient evaluation can contribute to early diagnosis, reducing avoidable hospitalizations and improving prognostic assessment [28,29,30,31].

In asthma, ML models (e.g., logistic regression, boosting, random forest) have been used to predict exacerbations using variables such as systemic steroid use, short-acting β_2_-agonist use, emergency department visits, age, and exacerbation history [6,32,33]. In parallel, wearable technologies coupled with AI have been explored to monitor physiologic signals and generate predictive alarms for impending asthma attacks, supporting earlier interventions [34]. In COPD, AI has also been applied to accessible modalities such as lung sounds (including DL-based approaches for disease detection/classification), supporting potential use in resource-limited settings [7,35,36,37]. In imaging-based assessment, radiomics combined with ML (e.g., SVM models) has been investigated to classify and stage COPD severity from chest CT features [8,38,39,40], and DL systems using CT imaging have shown potential for identification and staging while predicting clinically relevant outcomes (e.g., exacerbation risk, emphysema severity, spirometric obstruction, mortality) [12,41,42].

**Figure 2:**
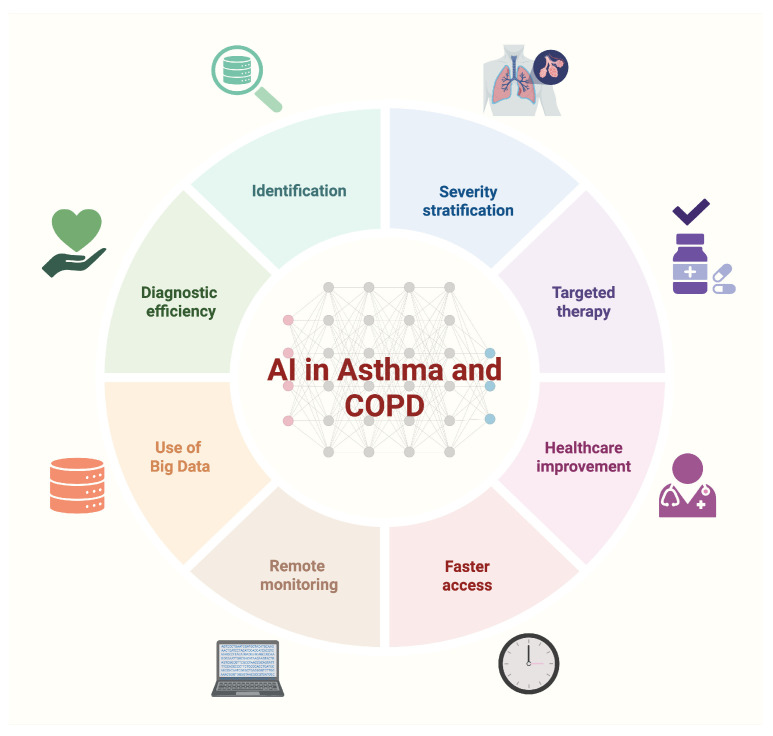
The use of AI in asthma and COPD, mapped to clinical tasks including differential diagnosis, severity stratification, phenotyping, outcome prediction (e.g., exacerbations/hospitalizations), and remote monitoring. Created in BioRender. Marrelli, F. (2026) https://BioRender.com/m3v3veq.

Figure 2 summarizes key domains in which AI has been applied along the clinical pathway in asthma and COPD, ranging from identification and severity stratification to targeted management and remote monitoring.

### 4.2. Role of Artificial Intelligence Techniques for Asthma Phenotyping

Asthma is a heterogeneous condition, and phenotyping/endotyping aims to identify clinically meaningful subgroups based on symptom patterns, lung-function impairment, and inflammatory traits to guide personalized therapy [43,44]. There is considerable promise in using AI to identify individuals at higher risk of developing asthma by integrating large-scale genetic data with routinely collected clinical information; similarly, ML can analyze medication histories and digital medical records to detect early signals that may be missed by clinicians [33]. AI and ML approaches are increasingly employed to classify patients according to disease severity and phenotype and to support monitoring strategies [1]. Clinical information, including medical history, symptom patterns, pulmonary function tests, and auscultated lung sounds, represents a major data source for asthma diagnosis, screening, and phenotype-related assessment [9].

In this context, respiratory sounds represent a scalable phenotype-related signal that can be captured outside the clinic. The primary aim of Oletic and Bilas’s wearable sensor was to identify wheeze by recording breathing sound signals and transmitting them to a smartphone after specific signal alterations. The accuracy, sensitivity, and specificity of the final model were 94.91%, 89.34%, and 96.28%, respectively [45].

The forced oscillation technique (FOT) provides effort-independent measurements of respiratory mechanics and can capture airway obstruction traits relevant to asthma phenotyping, particularly in children or in patients unable to perform reliable spirometry. Amaral et al. evaluated multiple ML approaches using FOT-derived parameters as input to detect airway obstruction in asthma, supporting the feasibility of oscillometry features as phenotype-relevant signals for ML-based assessment [46]. Unlike conventional lung function tests, FOT is non-invasive and has the advantage of not requiring forced breathing maneuvers, enabling repeated measurements and potential longitudinal monitoring [46].

Beyond respiratory sounds, physiology-based signals can further support phenotype-oriented assessment by capturing airway mechanics and ventilation dynamics that may not be fully reflected by symptoms alone.

Capnography yields a continuous CO_2_ waveform (capnogram) that reflects ventilation dynamics and expiratory flow characteristics. Singh et al. performed automatic quantitative analysis of the respired CO_2_ waveform and used a support vector machine classifier to differentiate asthma from non-asthma subjects, demonstrating the potential of capnogram-derived features as ML inputs for asthma-related classification [47]. However, the limited dataset and lack of broad external validation in this work prevent an adequate assessment of the proposed modality for routine phenotype-oriented assessment or monitoring [47].

The exhaled breath condensate (EBC) of 89 asthmatic patients and 20 healthy controls was examined, and a random forest classifier was developed to differentiate between the two groups. The final classifier exhibited 80% sensitivity and 75% specificity. Looking for non-invasive asthma biomarkers, the EBC could be another potential target. However, this technology requires better standardization before it can be implemented in broader clinical settings [48,49].

Regarding asthma classification and phenotypes, several scientists have developed an ML technique for identifying pediatric asthma phenotypes based on patients’ responses to controller drugs. Bronchodilator response and blood eosinophils have been identified as the strongest predictive indicators of asthma management in the pediatric group under study [10]. Asthma exacerbations are crucial to the disease’s progression and management, resulting in significant direct and indirect expenditures. Asthma models have been created to assess attack risk in real time, utilizing sensors that collect physiological and environmental information. The information obtained is sent to a smartphone and analyzed by a random forest classifier, which recognizes asthma attacks with an overall accuracy of 80.1% [34]. Determining whether hospitalization is necessary during an asthma attack is a crucial decision. The suggested gradient-boosting machine-based approach measures total risk and determines whether hospitalization is needed [50].

Overall, current asthma phenotyping work using AI falls into several reproducible patterns: treatment-response phenotypes, such as pediatric subgrouping based on response to controller medications [10]; biomarker-/omics-informed approaches, including exhaled breath condensate-based classifiers and breath biomarker frameworks that support non-invasive phenotype discovery, while highlighting the need for standardization [48,49]; and digital phenotyping, where respiratory sounds or wearable-derived signals provide measurable traits (e.g., wheeze detection) outside the clinic [45], complemented by physiology-based traits captured by oscillometry and capnography-derived waveform analysis [46,47]. In parallel, ML models have been developed for actionable decisions during exacerbations, such as estimating the need for hospitalization at emergency department triage [50]. Despite promising results, many studies remain limited by sample size, single-center design, and inconsistent external validation, which may affect generalizability and clinical reliability [1].

### 4.3. Role of Artificial Intelligence Techniques for COPD Classification

Radiology and CT are central to COPD characterization because they enable quantitative assessment of emphysema, airway remodeling, and extrapulmonary features that are clinically linked to outcomes. In this context, AI methods have been applied to automate CT-derived quantification and to support COPD diagnosis and severity stratification (“staging”), often by integrating imaging information with clinical variables such as spirometry to improve classification performance [8,12,38,39,40]. High-resolution CT (HRCT) provides detailed views of airway and parenchymal structure, and AI-driven analysis can reduce inter-reader variability and scale interpretation across large datasets [51,52].

CT can also capture clinically relevant COPD comorbidities and overlapping conditions. For example, bronchiectasis is detectable on CT in a substantial proportion of COPD patients and is associated with exacerbations and mortality [53]. In addition, CT-derived measures such as coronary artery calcium, pulmonary artery enlargement, bone density, and muscle mass provide prognostic information and are independently associated with all-cause mortality [54,55].

HRCT generates high-dimensional data, and radiomics can extract quantitative descriptors associated with COPD presence and severity [8,38]. These features can then be exploited by ML models to classify COPD from CT and to support severity stratification [39]. Notably, adding radiomic descriptors to an SVM classifier has been reported to reliably categorize COPD stages and to outperform prior approaches, supporting radiomics+ML as a viable pathway for CT-based COPD staging [40]. More broadly, AI methods can automate and optimize CT analysis once trained on appropriate datasets, improving consistency and throughput [51,52,53].

Beyond parenchymal radiomics, airway-structure features such as branching patterns, lengths, and wall thickness can further improve CT-based COPD characterization and may enhance ML performance when combined with original CT image features [56,57]. Examining the bronchial tree’s branching, lengths, and wall thicknesses may provide useful data [56]. It is possible to generate a full representation of the lung using representations of 3D airway trees and lung fields, for COPD diagnosis and combining these characteristics with the original CT image features, improving the performance of ML models in COPD [12,57].

Additionally, DL algorithms are used to identify and stage COPD: a specific DL system may recognize notable regional image characteristics from high-resolution CT scans of COPD patients, revealing strong predictive capabilities for exacerbation risk, emphysema severity, spirometric obstruction, and mortality, which may improve both research and clinical practice [12,41].

AI impacts emphysema diagnosis, subtyping, and phenotypes. Emphysema is defined as the irreversible destruction of the alveoli and it is typically classified into three subtypes based on CT scans distribution: centrilobular emphysema (CLE), prevalent in smokers; panlobular emphysema (PLE), often linked to α1-antitrypsin deficiency; and paraseptal emphysema (PSE). CT can assess the severity of emphysema using densitometric analysis, which quantifies the lung’s mean attenuation and the proportion of lung volume with low attenuation [12,58].

AI algorithms can assess the degree of emphysema by automatically estimating the compromised lung capacity, offering a more objective and consistent classification than human densitometry [59]. AI has improved the diagnosis of emphysema in several ways: enhanced sensitivity through ML and traditional features; utilizing DL to extract features automatically; convolutional neural networks demonstrate promising accuracy levels while reducing processing time, automatically identifying and isolating emphysematous regions without the need for manual feature engineering, and discriminating between subtypes of emphysema in high-resolution CT lung images [11,60]. Large patches clipped from CT images are employed for embedding and classification in a vision transformer (ViT) model for categorizing emphysema subtypes. The ViT model holds potential for additional medical applications and accurately classifies emphysema subtypes [61].

Automated CT algorithms could enhance COPD severity staging and provide prognostic insights by measuring emphysema and air trapping in CT images using a DL-based method [62]. CT also aids in the decision-making process for lung volume reduction surgery (LVRS) or endobronchial valve insertion. However, there is a growing inclination towards quantitative analysis of emphysema position, size, and fissure integrity to facilitate decision-making for endobronchial valve treatment. Emphysema is associated with an accelerated drop in forced expiratory volume in one second (FEV_1_), an elevated mortality risk, and an increased probability of lung cancer development [24].

Dosovitskiy et al. use the Transformer architecture for image categorization in the ViT model, which is useful for identifying different types of emphysema and may increase the precision and effectiveness of COPD assessment. The model uses data augmentation and transfer learning on a labelled dataset of CT scans with annotated emphysema subtypes to improve performance and generalizability [12,63].

Pang et al. suggested the Generative Adversarial Network (GAN) technique for producing contrast-enhanced or non-contrast CT images. A discriminator and a generator make up the network architecture of the suggested synthesizer. The generator can learn to create realistic contrast-enhanced (CE) or non-contrast (NC) CT scans, by using the discriminator to distinguish between real and fake images. In order to create a probability map, the NC CT and real CE CT are also combined and fed into the discriminator [64,65].

MIL is particularly relevant for COPD because CT labels are often available at the patient level (COPD vs. non-COPD, or severity groups), while disease patterns are regionally heterogeneous within the lung; MIL can therefore learn from weak labels by aggregating patch-level information [57,66]. Using CT scans, Multiple Instance Learning (MIL) can detect COPD. With the support of labelled bags and a large number of examples, the MIL paradigm facilitates learning of COPD patterns from weakly labelled data, such as the overall COPD status of each CT scan. A CT scan is represented by a bag, and the examples inside the bag stand in for different areas or patches of the picture. MIL can control the disease’s heterogeneity because it can detect COPD through local patterns or anomalies in particular areas of the CT scan. Patch extraction, feature extraction, attention-guided instance-level prediction, and bag-level aggregation are the steps needed to set up MIL-based COPD identification [57,66].

CNN aims to identify unique characteristics that represent regional variations and patterns linked to COPD in the feature extraction process. Airway thickness, the presence of mucus plugs, emphysema, and other anatomical changes in the lungs that signify disease can all be recognized by training Convolutional Neural Networks (CNNs). The degree of these alterations, which reflects the severity of the illness, can also be measured using CNNs [12,56,57]. The risk level of the population may be measured with the help of CNN analysis. A multilayer CNN was able to predict episodes of acute respiratory illness, smoker mortality, and accurately diagnose and stage COPD using only CT imaging data [42]. Events in attention-guided instance-level prediction are assigned varying weights according to their significance for COPD prediction. The attention process ignores the less significant portions while identifying and prioritizing the most instructive. Each instance is given an attention rating, which is then used to modify the instance-level predictions. Either adding attention layers to the MIL design or using a separate attention network, can produce an attention score. A bag-level evaluation that reflects the overall COPD state of the CT image is produced by combining the instance-level predictions. Max pooling, average pooling, and attention-based mechanisms are examples of common aggregation strategies [57,66].

The MIL framework has a number of advantages, including adaptability and the ability to be used with different aggregation algorithms and DL architectures to improve the precision and resilience of COPD detection [57,66]. In order to create a highly precise automatic algorithm for the severity of COPD, Ying et al. used a deep belief network, proving that it is a useful tool for determining the risk of exacerbations in patients with COPD [67].

Both pulmonary and extrapulmonary vascular alterations are associated with the pathophysiology of COPD. Submillimeter vascular changes can be detected by AI tools, improving our knowledge of vessel dynamics [68]. Various methods are employed, including transformer-based networks, CNN, and generative adversarial networks [69,70].

Overall, current work on AI in CT-based COPD classification spans radiomics-driven staging, DL-based identification and outcome prediction, the use of weakly supervised frameworks such as MIL to capture regional heterogeneity, and vessel-focused models to quantify pulmonary and extrapulmonary alterations [8,12,38,39,40,41,57,66,67,68,69,70].

Table 2 summarizes the main AI-driven applications investigated in asthma and COPD, reporting for each area the typical algorithms, input data sources, and the corresponding clinical purpose.

### 4.4. Future Perspectives of Artificial Intelligence in Asthma and COPD

**Figure 3:**
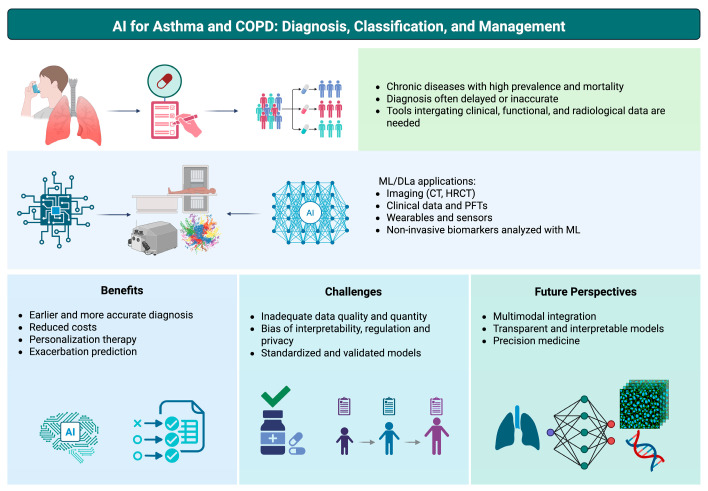
Conceptual overview of AI in asthma and COPD management: benefits, challenges/limitations, and future perspectives. Created in BioRender. Pelaia, C. (2026) https://BioRender.com/qy3blub.

The reviewed literature indicates that AI can support asthma and COPD across diagnosis, phenotyping/severity stratification, quantitative imaging, and outcome prediction, with particular strengths in processing high-dimensional data such as CT and longitudinal digital health streams [12,65,71,72,73]. However, the clinical usefulness of many proposed tools remains contingent on robust validation and demonstration of added value within real-world workflows. Key benefits, current challenges/limitations, and future perspectives of AI applications in asthma and COPD are summarized in Figure 3.

In COPD, AI-enabled CT analysis has shown promise for quantitative emphysema assessment, region segmentation, and severity stratification, while also enabling prediction of clinically meaningful outcomes (e.g., exacerbations and mortality) [12,41,42]. In parallel, lightweight approaches for COPD screening in primary care (e.g., simplified spirometry + mobile-enabled workflows) may improve early identification in underserved settings [65]. For asthma, integrating sensor data and sequential models (e.g., LSTM-based pipelines) may improve real-time risk prediction and proactive management [33,34].

Across both diseases, barriers to adoption include data quality and dataset shift, limited external validation, interpretability constraints, and privacy/regulatory requirements [2,12,33,74]. Under-representation of specific demographic or clinical subgroups may distort predictions and exacerbate bias, underscoring the need for diverse training cohorts and continuous monitoring after deployment [33]. Clear data regulations and governance frameworks are also necessary to ensure patient data ownership, access, sharing, and secure monitoring [74].

Improving clinical trust requires interpretable models and transparent reporting of model rationale (e.g., attention maps or clinically meaningful feature importance), together with integration into existing clinical pathways [2,12,57,71].

Table 3 summarizes future perspectives and key development priorities for AI in asthma and COPD management.

## 5. Conclusions

AI is expected to remain a prominent research hotspot in the coming years for the diagnosis and treatment of CRDs. Advanced algorithms expedite diagnosis, facilitating more efficient and tailored therapies. AI/ML methods are particularly beneficial when analyzing large, complex datasets that include information from various sources. Since asthma and COPD are chronic conditions that require monitoring over several years and whose symptoms can be identified at the cellular, organ, and organismal levels, they are ideal candidates for the application of AI/ML. Large-scale data must be analyzed in tandem, as environmental factors are crucial to the pathophysiology and natural history of asthma and COPD. To frame all potential CRD effects across scale and time, a theoretical study should ideally collect genomic, metabolomic, clinical, and environmental data from vast and heterogeneous patient cohorts across successive time slices. Asthma and COPD are common and treatable diseases, yet extensive underdiagnosis and misdiagnosis result in patients receiving no treatment or inappropriate treatment. Additionally, while they share similarities and comparable symptoms, these conditions also exhibit significant differences that should impact clinical decision-making. The ability of AI to create personalized health plans is evident, as it can offer customized solutions based on the patient’s history and current condition. This review provides an integrative synthesis of AI applications for asthma and COPD in outpatient care, highlighting potential clinical and economic benefits, the main implementation challenges (e.g., misdiagnosis risk, bias, data quality, interpretability, regulation and privacy), and the most relevant future development priorities to support safe translation into practice. Furthermore, AI’s transformative potential in clinical trials—particularly to enable decentralized participation and remote monitoring—holds significant promise. However, safe implementation requires addressing key challenges such as misdiagnosis risk, systematic errors, spurious associations, limitations in differential diagnostic criteria, and security and privacy vulnerabilities. Future investigations on AI for CRD management should concentrate on the following main goals: standardizing AI models and algorithms to guarantee consistent outcomes and facilitate broad clinical adoption; developing interpretable AI systems to bolster clinicians’ comprehension and confidence in AI-generated predictions; tackling data privacy concerns with stringent protocols to foster trust among patients and healthcare providers; and augmenting diverse datasets to enhance AI model robustness, thereby improving generalization and accuracy in diagnosing lung diseases. These collaborative efforts will strengthen effectiveness, transparency, and trust within healthcare environments. Despite the challenges posed by the complex ‘black box’ nature of some AI approaches and the need for rigorous training and validation, the future appears promising.

## Figures and Tables

**Table 1:** Summary of representative included studies: overview of study designs, sample sizes, and validation strategies.

Author, Year, Reference	Country	Study Design	Sample Size	Validation Strategies
Srivastava et al., 2021. [4]	India	Retrospective observational study	126 patients	Internal validation of the deep learning model
Spathis et al., 2019. [5]	Greece	Retrospective observational study	132 patients	Train/Test Split, Cross-Validation (5-fold and 10-fold) and standard performance metrics
Zein et al., 2021. [6]	USA	Retrospective observational cohort study	60,302 patients	Train/test split, 5-fold cross-validation and standard performance metrics such as AUC, precision and F1 score
Altan et al., 2020. [7]	Turkey	Retrospective observational study	41 patients	Training set/test set, k-fold cross-validation, accuracy, precision, F1 score, compared with clinical standard.
Yang et al., 2022. [8]	China	Retrospective observational study	468 patients	Training/test split, internal cross-validation and ROC metrics
Kaur et al., 2018. [9]	USA	Cross-sectional study nested in a birth cohort study	427 patients	Manual review of medical records, separate training/test sets, and standard diagnostic metrics to evaluate the accuracy of the NLP model
Ross et al., 2018. [10]	USA	Retrospective observational cohort study	1688 patients	Training/test sets, internal cross-validation, standard predictive metrics, and comparison with traditional methods
Karabulut et al., 2015. [11]	Turkey	Retrospective observational study	93 patients	Training/test split, cross-validation and comparison with clinical assessments

**Table 2:** Current Applications of Artificial Intelligence in the Diagnosis and Characterization of Asthma and COPD.

Application Domain	AI/ML Technique Used	Purpose/Function	Input Data	Key Results/Performance	Limitations
Diagnosis and Differential Diagnosis	Logistic Regression, Random Forest, Boosting [1,6,19].	Differentiate asthma from COPD and predict exacerbations.	Clinical data (age, smoking history, symptoms) and spirometry [5,6,19].	Identification of key predictors for exacerbations (e.g., steroid use) [1,6].	Risk of inaccurate diagnosis; misclassify asthma vs. COPD; reducing reliability and reproducibility [1,5,6,19].
Asthma Phenotyping	Random Forest [10].	Identify pediatric phenotypes and classify patients based on treatment response.	Response to controller medications, blood eosinophils, bronchodilator test [10].	Identification of predictive biomarkers for personalized therapy [10,43,44].	Heterogeneous or incomplete data; small or unrepresentative cohorts; reduced interpretability; challenging to apply clinically.
COPD Classification from Imaging	Support Vector Machine (SVM) with Radiomics [40].	Stage COPD severity.	Radiomic features extracted from chest CT scans [8,38,40].	Accurate classification of COPD stages, outperforming existing methods [40].	Limits generalisability; affects model consistency; risk of overfitting; reduced interpretability of models.
Wheezing and Respiratory Sound Detection	Deep Learning Algorithms [4,7,45].	Detect wheezing and classify COPD severity from lung sounds.	Respiratory sound recordings (12-channel auscultation or wearable sensors) [7,45].	Accuracy > 94% in wheezing detection [45] and COPD classification (AUC 0.965) [7].	Reduced generalizability and interpretability; sensitivity to environmental noise and microphone quality; difficulty distinguishing wheezing from other respiratory sounds.
Lung Structure Analysis (Emphysema)	Convolutional Neural Network (CNN), Vision Transformer (ViT) [11,12,42,61].	Quantify, segment, and classify emphysema subtypes.	High-resolution CT (HRCT) scans [12,58].	Automation of quantification and accurate classification of emphysema subtypes (CLE, PLE, PSE) [11,59,61].	Limited generalisability and external validation; reduced interpretability; indirect clinical correlation.

**Table 3:** Future Perspectives on and the Development Potential of Artificial Intelligence in the Management of Asthma and COPD.

Future Development Area	Potential Application	Related Technologies/Challenges	Expected Impact
Integrated Precision Medicine	Combine imaging (CT), genomic, proteomic, and environmental sensor data for holistic predictive models [12,33,73].	Data integration challenges, interoperability, governance, and privacy [2,74].	Identification of endotypes, prediction of treatment response, and discovery of new drug targets [12,43,44].
Dynamic and Real-Time Predictive Models	Develop 4D models to visualize airway dynamics and predict exacerbations in real time [12,33,34].	Integration with wearable sensor data (IoT), sequential AI models (e.g., LSTM) [33,34].	Timely preventive interventions, reduction in hospitalizations, and proactive disease management [1,33].
Interpretability (XAI) and Clinical Reliability	Develop “Explainable AI” (XAI) models to clarify the rationale behind AI decisions [12,71].	Development of algorithms for attention maps and clinically relevant feature importance [12,57].	Increased clinician trust, easier implementation into routine clinical practice [2,12,71].
Early Screening and Primary Care Diagnostics	Implement lightweight algorithms for COPD screening in primary care settings [65].	Development of mobile apps, analysis of basic clinical data and simplified spirometry [65].	Early diagnosis, reduced underutilization of resources, and access to care in underserved populations [37,65].
Clinical Trial Optimization	Identify and enroll patients for clinical trials by screening electronic databases [9].	Natural Language Processing (NLP) for medical records, identification of digital phenotypes [9].	Accelerated recruitment, more efficient trials, and development of more targeted therapies [72].

## Data Availability

No new data were created or analyzed in this study.

## Abbreviations

AI	Artificial Intelligence
CNN	Convolutional Neural Network
COPD	Chronic Obstructive Pulmonary Disease
CRDs	Chronic Respiratory Diseases
CT	Computed Tomography
DL	Deep Learning
EBC	Exhaled Breath Condensate
FOT	Forced Oscillation Technique
GAN	Generative Adversarial Network
LVRS	Lung Volume Reduction Surgery
MIL	Multiple Instance Learning
ML	Machine Learning
NLP	Natural Language Processing
SVM	Support Vector Machine
HRCT	High-Resolution Computed Tomography
PFT	Pulmonary Function Test
FEV_1_	Forced Expiratory Volume in 1 s
AUC	Area Under the Curve
ViT	Vision Transformer
CLE/PLE/PSE	Centrilobular/Panlobular/Paraseptal Emphysema
IoT	Internet of Things
LSTM	Long Short-Term Memory networks
